# MCOIN: a novel heuristic for determining transcription factor binding site motif width

**DOI:** 10.1186/1748-7188-8-16

**Published:** 2013-06-27

**Authors:** Alastair M Kilpatrick, Bruce Ward, Stuart Aitken

**Affiliations:** 1 School of Informatics, University of Edinburgh, Informatics Forum, 10 Crichton Street, EH8 9AB Edinburgh, Scotland; 2 School of Biological Sciences, University of Edinburgh, Darwin Building, King’s Buildings, Mayfield Road, EH9 3JR Edinburgh, Scotland

**Keywords:** Transcription factor binding sites, Motif discovery, Bacterial motifs, Motif width

## Abstract

**Background:**

In transcription factor binding site discovery, the true width of the motif to be discovered is generally not known *a priori*. The ability to compute the most likely width of a motif is therefore a highly desirable property for motif discovery algorithms. However, this is a challenging computational problem as a result of changing model dimensionality at changing motif widths. The complexity of the problem is increased as the discovered model at the *true* motif width need not be the most statistically significant in a set of candidate motif models. Further, the core motif discovery algorithm used cannot guarantee to return the best possible result at each candidate width.

**Results:**

We present MCOIN, a novel heuristic for automatically determining transcription factor binding site motif width, based on motif containment and information content. Using realistic synthetic data and previously characterised prokaryotic data, we show that MCOIN outperforms the current most popular method (E-value of the resulting multiple alignment) as a predictor of motif width, based on mean absolute error. MCOIN is also shown to choose models which better match known sites at higher levels of motif conservation, based on ROC analysis.

**Conclusions:**

We demonstrate the performance of MCOIN as part of a deterministic motif discovery algorithm and conclude that MCOIN outperforms current methods for determining motif width.

## Introduction

Recent advances in biology have led to a huge increase in the amount of data available for study. Of considerable interest to biologists are transcription factor binding site (TFBS) motifs; short DNA sequence patterns that have important roles in gene transcription and regulation. Discovery and further analysis of these sequences remains an important task in the wider challenge of understanding the mechanisms of gene expression (examples from the recent ENCODE project include [1-3]). Consequently, there is much continuing interest in developing algorithms which can automatically discover TFBS motifs [4].

Automatically determining the width of a novel TFBS motif is a desirable property for motif discovery algorithms since the true motif width is generally not known *a priori*. An ideal algorithm would be executed over a range of reasonable candidate widths and return the most likely result based on some criterion. This is an important but challenging computational problem, as the likelihood function maximised by motif discovery algorithms cannot be used directly to compare models with different motif widths [5]. The difficulty partially stems from the fact that the maximum value of the joint likelihood of the model given the data and the missing information is bound to increase with increasing motif width as a consequence of the increasing number of free parameters [5-7]. The complexity of the problem is increased when additional constraints on the parameters (e.g. the palindrome constraint in the popular MEME algorithm) are employed, as the maximum likelihood value of models with parameter constraints will be lower than unconstrained models of the same motif width. To some degree, this problem corresponds to the more general problem of model selection in statistics. A number of general model selection criteria which incorporate adjustments for model dimensionality have been used in other areas with success [8,9]. However, these criteria have generally not performed well at determining motif width in known datasets [5].

**Figure 1:**
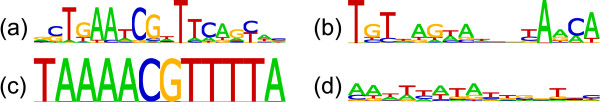
**Diversity of *E. coli *motifs.** Sequence logos for four *E. coli* motifs illustrate the diversity of motifs in terms of information content profile. **(a)** FruR has a number of perfectly conserved positions in the centre of the motif, flanked by positions which are less well-conserved. **(b)** The gapped motif of DeoR illustrates the opposite: two well-conserved segments are separated by an unconserved ‘gap’. **(c)** All positions in the MalI motif are perfectly conserved. **(d)** The Nac motif has few well-conserved positions.

**Figure 2:**
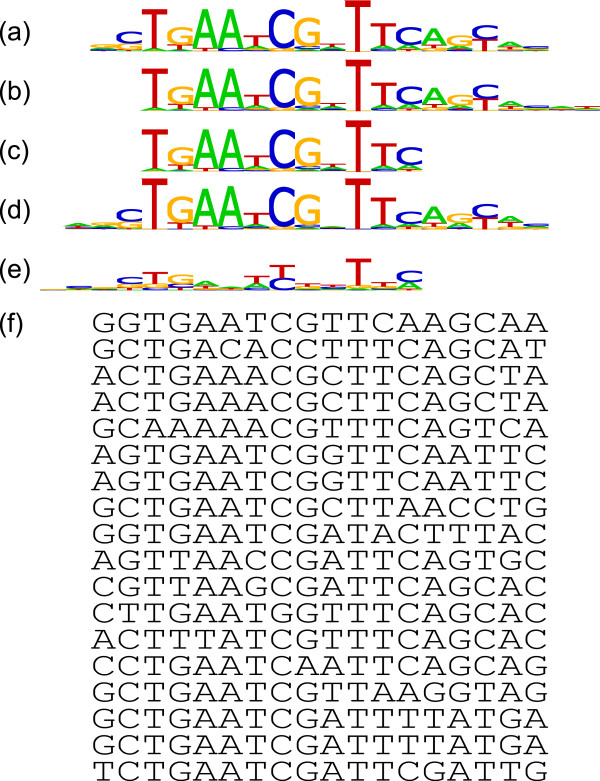
***E. coli *FruR motif sequence logos and occurrences.(a-e)** Known and inferred E. coli FruR motif sequence logos. (a) The known E. coli FruR motif. The central part of the motif has a number of well-conserved positions; however, the outermost positions are very poorly conserved and may be incorrectly statistically regarded as background. A heuristic for determining the most likely width is required to be robust in statistically unclear situations such as this. (b) A motif discovery algorithm may become locked in a non-optimal local maximum of the likelihood function which corresponds to a shifted version of the true motif. (c) The most statistically significant model in a set of candidate models may only represent a portion of the true motif. (d) From the candidate set of computationally discovered models, MCOIN chooses the model at w^**∗**^ + 1, which corresponds well with the true motif. (e) The E-values estimator chooses the model at w^**∗**^ - 3, which corresponds less well with the true motif. (f) Known occurrences of the E. coli FruR motif.

The complexity of the computational problem is further increased by the diversity of TFBS motifs (Figure 1). Clearly, biologists are interested in the true motif width; however, while some motifs provide statistically strong signals, the majority of motifs are more subtle and in the worst cases may be statistically indistinguishable from random artefacts in a given set of DNA sequences [4]. This subtlety means that the most statistically significant motif width need not match the biologically known true motif width. As an example, the true width of the FruR motif in *E. coli* is known to be 18bp. However, the sequence logo and known FruR binding sites (Figure 2) show that the outermost motif positions are very poorly conserved, providing little information above that of background ‘noise’. Furthermore, motif discovery algorithms cannot guarantee to return the best possible result at each candidate width. Such algorithms often display a phenomenon known as ‘shifting’ (Figure 2), where a motif is only partially recovered, along with some additional non-motif ‘background’ positions [5]. This is in part due to the above fact that, from a statistical viewpoint, the true boundaries of a motif are often unclear. Although strategies to deal with this phenomenon have been devised (for instance, GMA in [10]), none can provide a guarantee that shifting is completely eliminated. This means that, even if the true motif width were known in advance, a motif discovery algorithm is not guaranteed to discover this motif perfectly. We therefore require a heuristic which is robust in practice, coping with both cases where a statistically strong motif signal is present and where the motif signal is more subtle.

Attempts at a heuristic to automatically determine motif width in a deterministic (Expectation-Maximization, or EM-based) algorithm have included functions based on the maximum likelihood ratio test (LRT) [11], methods based on V -fold cross-validation [7] and the Bayesian Information Criterion (BIC) [12]. However, in practice, estimators based on the E-value of the resulting multiple alignment are used instead [4]. The E-value of the multiple alignment of predicted motif occurrences is an approximate p-value for testing the hypothesis that the predicted motif occurrences were generated from the predicted model against the null hypothesis that the predicted occurrences were generated by the background model. Typically, E-values are calculated for models at each candidate width and the model with the minimum E-value chosen.

Here, we validate a novel heuristic for automatically determining the width of a motif in deterministic motif discovery algorithms, based on motif containment and information content (MCOIN). Based on tests with previously characterised prokaryotic TFBS motifs, we show that MCOIN outperforms the E-value of the resulting multiple alignment as a predictor of motif width, using mean absolute error. MCOIN is also shown to improve the overall correctness of results, based on receiver operating characteristic (ROC) analysis. Finally, we show that the performance of MCOIN will improve as the performance of the core motif discovery algorithm improves.

## Approach

The MCOIN heuristic is based on the concepts of motif containment and mean information content per column. If it is assumed that the motif discovery algorithm discovers the true motif within the dataset as well as possible at every candidate width {*w*_*min*_*,…,w*_*max*_}, then the algorithm discovers the true motif *exactly* at the *true* width *w*^∗^. It follows that, at candidate widths smaller than the true width (that is, {*w*_*min*_*,…,w*^∗^ - 1}), only a portion of the true motif is discovered while at candidate widths larger than the true width (that is, {*w*^∗^ + 1,…,*w*_*max*_}), the full motif is discovered, along with a number of background positions. Clearly, these models must be similar and are describing the same underlying motif. If we know that the models for widths w-1 and w are describing the same motif and also assume that model selection criteria (e.g. BIC) will choose the shorter model due to it having fewer free parameters, then the model with width w-1 can be removed from the set of candidate models as the width-w model also describes the same motif.

Retaining the assumption that the motif discovery algorithm discovers the true motif as well as possible at every candidate width, it follows that the model at the true width *w*^∗^ will also be removed as a result of it being contained within the model at width *w*^∗^ + 1. The result of discarding models based only on containment would be to discard all but the longest model. Clearly, we would prefer models at widths *w*_*min*_ to *w*^∗^ - 1 to be discarded in favour of the model at width *w*^∗^, but this model not to be discarded in favour of longer models. Calculating the mean information content per column (IC/col) for each model allows a method of stopping containment at widths greater than *w*^∗^. If, for example, the IC/col of the model at width *w*^∗^ is B bits, the model at width *w*^∗^ + 1 will have these same columns plus an additional background column, which will have a very low information content (if each nucleotide in the background model is equiprobable, the information content of this column will be 0 bits); the low information content of this additional background column will make the IC/col of the model at *w*^∗^ + 1 less than B bits. We can therefore modify our model selection process, discarding a shorter model in favour of a longer model only if the shorter model is contained within the longer model and the IC/col of the longer model is similar to that of the shorter model.

At a high level, this is implemented as follows: the position weight matrix (PWM; the probabilistic model of a motif used in motif discovery algorithms) of the shortest model (*w*_*min*_) is tested against each longer model (*w*_*min*_ + 1,…,*w*_*max*_), calculating the mean root Jensen-Shannon divergence per column (JSD/col) for each comparison. The Jensen-Shannon divergence [13] is used a measure of similarity; intuitively, the lower the JSD/col, the more similar the PWMs are. The IC/col ratio of the longer model to the shorter model is then calculated. If this is significantly lower than 1, we can assume that the additional column in the longer model is not information-rich and the longer model is longer than the true motif width. If the shorter model is ‘contained’ within the longer model (that is, the minimum JSD/col is smaller than some similarity threshold *t*_*sim*_, where 0 ≤ ***t***_*sim*_ ≤ 1) *and* the models have similar information (that is, the IC/col ratio of the longer model to the shorter model is greater than some information threshold *t*_*info*_), the shorter model is removed from the set of candidate models. The process is repeated for model widths *w*_*min*_ + 1 to *w*_*max*_ - 1 (the longest model is always kept in the set of candidate models). The remaining model with the lowest BIC score is chosen as our best estimate of motif width.

## Method

We assume throughout that we have a dataset X consisting of all overlapping width-w subsequences {*x*_1_,…,*x*_*n*_} from a number of DNA input sequences, as described by Bailey and Elkan [11]. We model X using a two-component mixture (TCM) model; such a model allows any number of non-overlapping motif occurrences within an input sequence. We further assume that we have a motif discovery algorithm which can predict a model ϕ = {θ,λ}, where θ={θ_0_,θ_1_} represents the background (θ_0_) and motif (θ_1_) models and λ represents the prior probability that a given position within the input sequences is a motif occurrence. From these, a log-odds scoring matrix LO and threshold t may be calculated:

(1) $$LO_{j,k}=\text{log}\;\left(\frac{f_{j,k}}{f_{0,k}}\right)$$

(2) $$t=\text{log}\;\left(\frac{1-\lambda }{\lambda }\right)$$

Together, *LO* and *t* form a Bayes-optimal classifier; each *x*_*i*_ is scored (using Equation 3) and deemed to be a motif occurrence if s(*x*_*i*_) > *t*[11].

(3) $$s(x_{i})=\overset{w}{\underset{j=1}{\sum }}\overset{T}{\underset{k=A}{\sum }}LO_{j,k}\;I(k,x_{i,j}),$$

where *I*(*k,a*) is an indicator function which is 1 if and only if *a* = *a*_*k*_ and 0 otherwise and *x*_*i,j*_ is the nucleotide in the *j*th position of sample *x*_*i*_. Let *x*_*pred*_ be the set of non-overlapping predicted motif occurrences and *n*_*pred*_ be the number of predicted motif occurrences |*x*_*pred*_|.

### Calculating the BIC for candidate models

We run our motif discovery algorithm over a number of reasonable candidate widths and return a model ϕ = {θ,λ} for each width. We assume that the unknown true motif width *w*^∗^ is within the range of tested candidate widths, that is, *w*_*min*_ ≤ *w*^*∗*^ ≤ *w*_*max*_.

For each width w ∈{*w*_*min*_,…,*w*^∗^,…,*w*_*max*_}, we use ϕ^*(w)*^ to create a set of predicted sites *x*_*pred*_, as described above. For each width, the log likelihood of a particular model ϕ^*(w)*^ given the predicted sites can be calculated:

(4) $$\;\text{log}\;L(\theta ,\lambda |x_{\text{pred}})=\overset{n_{\text{pred}}}{\underset{i=1}{\sum }}\text{log}\;\left[p(x_{i}|\theta_{1})\lambda +p(x_{i}|\theta_{0})(1-\lambda )\right],$$

where we define the distributions for the motif and the background model (following [11]) as:

$$p(x_{i}|\theta_{1})=\overset{w}{\underset{j=1}{\prod }}\overset{T}{\underset{k=A}{\prod }}{f_{\text{jk}}}^{I(k,x_{i,j})}$$

and

$$p(x_{i}|\theta_{0})=\overset{w}{\underset{j=1}{\prod }}\overset{T}{\underset{k=A}{\prod }}{f_{0k}}^{I(k,x_{i,j})}.$$

Following [9], we calculate the BIC for each model using:

(5) $$-2\text{log}\;L(\theta ,\lambda |x_{\text{pred}})+M\cdot \text{log}\;(n_{\text{pred}}),$$

where M is the number of free parameters in the model, equivalent to 3(w + 1). We now have a set of models$\left\{\phi^{\left(w_{\text{min}}\right)},\ldots ,\phi^{\left(w^{*}\right)},\ldots ,\phi^{\left(w_{\text{max}}\right)}\right\}$; each model with its own BIC score, based on its log likelihood (calculated using its set of predicted sites) and the number of model parameters. We now apply MCOIN, as described in the next section.

### MCOIN heuristic

Following [13], we first define the Jensen-Shannon divergence for two discrete probability distributions p and q as:

(6) $$\text{JS}(p||q)=\frac{1}{2}\text{KL}(p||m)+\frac{1}{2}\text{KL}(q||m),$$

where$m=\frac{1}{2}(p+q)$*** and ***$\text{KL}(p||q)=\underset{i}{\sum }p(i)\frac{p(i)}{q(i)}$. We also define the mean information content per column of a given motif model θ_1_ as:

(7) $$\text{IC}/\text{col}(\theta_{1})=\frac{1}{w}\overset{w}{\underset{j=1}{\sum }}\overset{T}{\underset{k=A}{\sum }}f_{j,k}{\text{log}}_{2}\left(\frac{f_{j,k}}{f_{0,k}}\right).$$

MCOIN relies on two threshold parameters, *t*_*sim*_ and *t*_*info*_. The value of *t*_*sim*_ may be chosen to be anywhere between 0 and 1. Choosing a good value for *t*_*sim*_ is important. If this value is too small, smaller models are required to match longer models more exactly before being discarded. Therefore, fewer models are discarded and MCOIN tends to choose models of shorter widths, leading to an underestimation of the true motif width. In contrast, if the value of *t*_*sim*_ is too large, shorter models may be discarded in favour of longer models when they are dissimilar, leading to an overestimation of true motif width. The optimal value of *t*_*sim*_ was calculated using tests on the realistic synthetic data collection described in the Data section; root mean squared error was minimised at *t*_*sim*_ = 0.32. Tests using the previously characterised E. coli data described in the Data section validated this parameter value: root mean squared error was minimised when 0.30≤*t*_*sim*_ ≤ 0.32. We therefore recommend *t*_*sim*_ = 0.32; this is intuitively reasonable as we would prefer to keep the value of *t*_*sim*_ low in order to ensure that two models are reasonably similar before discarding the shorter in favour of the longer. Tests which removed the motif discovery phase of the algorithm showed that the mean information content per column ratio alone was sufficient to choose the true motif width. That is, the value of *t*_*sim*_ had no effect. From this, we may conclude that, as motif predictions become stronger, the exact value of *t*_*sim*_ becomes less important. However, at current motif discovery algorithm performance levels, a value of 0.32 gives optimal results. It may be possible to change this value data-adaptively, but so far results have not shown this to be required.

We calculate *t*_*info*_ based on a perfectly conserved motif model having a mean information content per column of 2 bits. We define the ‘best case’ background column as having an information content of 1 bit (equivalent to a PWM column such as (0.5,0.5,0.0,0.0)^*T*^, where any two nucleotides are split equally). It is then possible to calculate the ‘best case’ IC/col ratio between two models of any given widths. If the actual IC/col ratio is less than the calculated ‘best case’, we deem the longer model to have unwanted background positions and do not discard the shorter model in favour of the longer model. The following example illustrates how the ‘best case’ IC/col ratio is calculated.

The calculation for the information threshold *t*_*info*_ can be generalised as:

(8) $$t_{\text{info}(w_{1}||w_{2})}=\frac{2w_{1}+(w_{2}-w_{1})}{2w_{2}},\;w_{2}>w_{1}$$

This is equivalent to adding the required number of columns *w*_*2*_ - *w*_*1*_ at 1 bit/col. MCOIN is outlined below.

### E-value of the resulting multiple alignment

The E-value of the multiple alignment of predicted motif occurrences [14] is an approximate p-value for testing the hypothesis that the predicted motif occurrences were generated from the predicted model against the null hypothesis that the predicted occurrences were generated by the background model. The E-value is then an estimate of the expected number of multiple alignments with statistical significance as great or greater than the observed alignment. Briefly, the E-value is calculated by computing the log-likelihood ratio of each column of the resulting multiple alignment of predicted sites and computing the p-value for each. The p-value of the product of column p-values is computed and then multiplied by the number of possible ways to select positions for the given number of sites in the set of input sequences to give the E-value. The E-value is calculated for models at each candidate width and minimised to select the best estimate of motif width [4,12].

### Data

#### ***Realistic synthetic data***

Five data collections, each consisting of 1,000 datasets, were created in order to test MCOIN. Each dataset contained 20 input sequences of length 200bp. Input sequences were created by extracting 200bp from the EcoGene [15] database of E. coli intergenic sequences, representing ‘background’ positions. Datasets were created so that each data collection had different mean levels of motif conservation, ranging from 0.51 to 2.00 bits/col: Motif positions within each sequence were chosen at random and a synthetic motif inserted. Synthetic motifs were created by choosing nucleotides (A, C, G, T) at random and randomly mutating positions in the motif occurrences so that the levels of conservation at each position could be controlled. Motif width was chosen to be 12 bp each time. A comparison of methods for determining motif width in [12] used datasets containing real (human) motifs with a minimum mean information content of 0.76 bits/col; the realistic synthetic data used in this study contains many motifs at lower levels of motif conservation, as analysis of known E. coli TFBS motifs indicated that significant numbers of motifs had mean conservation levels of less than 0.76 bits/col.

#### ***E. coli data***

Twenty datasets incorporating known E. coli TFBS sequences were created (Table 1). Background sequences were created as for realistic synthetic data. Positions within each 200 bp input sequence were chosen at random and a known TFBS sequence inserted. Known E. coli TFBS sequences were extracted from RegulonDB [16] for insertion in the background positions. We were concerned not to reproduce computationally predicted results. TFBS data from RegulonDB is supported by literature with experimental evidence; in the majority of cases this evidence stems from classical experimentation such as DNA footprinting and/or site mutation expression analysis and is not supported solely by human or computational inference. The experimental evidence for the data sequences used in this study is provided in Additional file 1.

**Table 1:** ***E. coli ***motifs

High conservation			Low conservation		
Name	*w*^∗^	*N*	**Name**	***w***^**∗**^	***N***
Ada	13	4	ArgR	18	35
CaiF	16	8	DeoR	16	7
CueR	19	3	FruR	18	18
IlvY	21	4	Fur	19	99
LacI	21	3	GntR	20	17
MalI	12	2	MalT	10	20
MelR	18	11	Nac	15	18
MetR	13	7	RcsB	14	11
PurR	16	20			
SoxR	19	2			
TorR	10	10			
XylR	18	4			

Summary of known*E. coli* TFBS motifs used in tests with real data. For each motif, transcription factor name is given, along with known width (*w*^x∗^, bp) and number of motif occurrences (*N*).

The number of motif occurrences in RegulonDB defined the number of input sequences; the mean number of input sequences was 15, ranging from 2 to 99 input sequences. The median number of input sequences was 9. Using known motif occurrences allows realistic motif conservation. The mean motif conservation was 1.13 bits/col, ranging from 0.49 to 2.00 bits/col. The median motif conservation was 1.04 bits/col. The mean motif width was 16bp, ranging from 10 to 21bp. The median motif width was 17 bp.

The data collection was split into two groups based on mean information content per column. The split was made at a value of 1 bit/col, producing a ‘high conservation’ group containing 12 datasets and a ‘low conservation’ group containing 8 datasets. In the ‘high conservation’ group, the mean number of input sequences was 7, ranging from 2 to 20 input sequences. The median number of input sequences was 4. The mean motif conservation was 1.36 bits/col, ranging from 1.02 to 2.00 bits/col. The median motif conservation was 1.31 bits/col. The mean motif width was 16bp, ranging from 10 to 21bp. The median motif width was 17bp. In the ‘low conservation’ group, the mean number of input sequences was 28, ranging from 7 to 99 input sequences. The median number of input sequences was 18. The mean motif conservation was 0.78 bits/col, ranging from 0.49 to 0.99 bits/col. The median motif conservation was 0.79 bits/col. The mean motif width was 16bp, ranging from 10 to 20bp. The median motif width was 17bp. Table 1 illustrates some of the diversity within the chosen*E. coli* motifs. The sequence logos of selected motifs (Figure 1) illustrate this further.

#### ***Prokaryotic ChIP data***

Nine datasets incorporating known prokaryotic motifs discovered by ChIP methods were also created (Table 2). Motifs from diverse species including*E. coli*[17-20],*V. cholerae*[21,22],*M. tuberculosis* [23,24] and*B. subtilis* [25] were used. Background sequences for the*E. coli* datasets were created as above. Background sequences for other species were created by randomly choosing nucleotides, altering the weighting to reflect GC-content as required. Again, positions within each 200 bp input sequence were chosen at random and a known TFBS sequence inserted.

**Table 2:** Prokaryotic ChIP motifs

**Species**	**Name**	***w***^**∗**^	***N***
*E. coli*	CRP	22	34
*E. coli*	LexA	20	25
*E. coli*	PurR	16	28
*E. coli*	RutR	16	19
*V. cholerae*	Fur	21	55
*V. cholerae*	RpoN	15	37
*M. tuberculosis*	DosR	18	24
*M. tuberculosis*	LexA	18	23
***B. subtilis***	Spo0A	12	94

Summary of known prokaryotic TFBS motifs used in tests with real data. For each motif, the species and transcription factor name is given, along with known width (*w*^∗^, bp) and number of motif occurrences (*N*).

The number of motif occurrences for each motif defined the number of input sequences; the mean number of input sequences was 38, ranging from 19 to 94 input sequences. The median number of input sequences was 28. Again, using known motif occurrences allows realistic motif conservation. The mean motif conservation was 0.99 bits/col, ranging from 0.56 to 1.25 bits/col. The median motif conservation was 1.04 bits/col. The mean motif width was 16bp, ranging from 10 to 22bp. The median motif width was 16 bp.

### Measuring performance

The performance of the heuristic on a data collection is assessed through its mean site-level sensitivity (*sSn*), mean site-level positive predictive value (*sPPV*) and the area under the receiver operating characteristic (ROC) curve (AUC). Following [26], we define a predicted site as a ‘true positive’ result if it overlaps the true site by at least a quarter of the true width.*sSn* (also known as*recall* in machine learning literature) measures the proportion of true positive sites which are correctly predicted as such.*sPPV* (also known as*precision*) measures the proportion of predicted positive sites which are actually true positives. For our purposes,*sSn* is defined as the fraction of true sites which are predicted and*sPPV* is defined as the fraction of predicted sites which are known to be true (see also [27]); that is:$\text{sSn}=\frac{\text{sTP}}{\text{sTP}+\text{sFN}}$ and$\text{sPPV}=\frac{\text{sTP}}{\text{sTP}+\text{sFP}}$.

AUC is the integral of the ROC curve plotting*sSn* against the site-level false positive rate ($\text{sFPR}=\frac{\text{sFP}}{\left(\text{sFP}+\text{sTN}\right)}$). The ROC curve is constructed by computing the probability of each possible site being an occurrence of the motif*p*(*Z*_*ij*_ = 1|*X*_*i*,*j*_,*θ*) and ranking each possible site based on this value.*sSn* and*sFPR* are plotted for all possible thresholds of*p*(*Z*_*i*,*j*_ = 1|*X*_*i*,*j*_,*θ*) and AUC calculated using the trapezoid rule. This is implemented using the ROCR R package [28].

While the above classification statistics provide an indication of how well the predicted sites associated with a motif model match the true sites, they give no indication of how well a heuristic estimates motif width. This performance is assessed here through the mean absolute error (MAE) and root mean squared error (RMSE), comparing the predicted motif width to the known width. RMSE is a commonly used measure but tends to exaggerate the effect of estimations which are further from the true value; in contrast, MAE treats all error sizes equally according to their magnitude. In most practical situations, the best estimator remains the best regardless of which error method is used [29].

## Results and discussion

In general, mean site-level sensitivity (*sSn*) and positive predictive value (*sPPV*) decrease with decreasing motif conservation. The decrease in*sSn* is a result of the motif discovery algorithm predicting fewer sites overall. That is, at lower motif conservations, fewer sites score highly enough such that*s*(*x*_*i*_) > *t* (see Equation 3). This leads to an increase in the number of false negative results (sites incorrectly classified as ‘background’) and therefore a decrease in*sSn*. The decrease in*sPPV* is attributable to background sites better matching the weaker motif model; as the model becomes weaker, the difference in scores between true motif occurrences and spurious background sites decreases. This can lead to an increase in the number of false positive results (sites incorrectly classified as motif occurrences) and therefore a decrease in*sPPV*.

### Width determination without discovery

MCOIN was initially evaluated without the motif discovery phase of the algorithm. That is, for each realistic synthetic dataset, the heuristic was tested using a set of candidate models which were constructed as if the motif discovery algorithm had discovered the motif in that dataset as well as possible at each candidate width. For each dataset, all candidate widths from*w*^∗^ - 4 to*w*^∗^ + 4 were tested. MCOIN is compared against the E-values estimator and also (following [12]) evaluations using the known width (equivalent to having a set of candidate models consisting only of*w*^∗^). Results of these evaluations are summarised in Tables 3 and 4.

**Table 3:** Tests without motif discovery: classification-based results

**Conservation**	**Known width (*w***^**∗**^)			**MCOIN (*w***^**∗**^** ± 4)**			**E-values (*w***^**∗**^** ± 4)**		
**(mean bits/col)**	***sSn***	***sPPV***	**AUC**	***sSn***	***sPPV***	**AUC**	***sSn***	***sPPV***	**AUC**
**2.00**	1.00	1.00	1.00	1.00	1.00	1.00	1.00	1.00	1.00
**1.49**	0.98	0.94	1.00	0.98	0.94	1.00	0.97	0.93	1.00
**1.08**	0.80	0.93	1.00	0.80	0.93	1.00	0.82	0.79	1.00
**0.76**	0.49	0.89	0.99	0.49	0.89	0.99	0.56	0.71	0.99
**0.51**	0.23	0.79	0.99	0.23	0.79	0.99	0.23	0.77	0.98

Mean site-level sensitivity (*sSn*), positive predictive value (*sPPV*) and area under the ROC curve (AUC) for five collections of realistic synthetic data at varying levels of motif conservation. In these tests, the motif discovery phase of the algorithm was removed and the set of candidate models constructed as if the motif discovery algorithm had performed as well as possible at each candidate width.

**Table 4:** Tests without motif discovery: mean error in motif width

**Conservation**	**MCOIN (*w***^**∗**^** ± 4)**		**E-values (*w***^**∗**^** ± 4)**	
**(mean bits/col)**	**MAE**	**RMSE**	**MAE**	**RMSE**
**2.00**	0.00	0.00	0.00	0.00
**1.49**	0.00	0.00	0.12	0.50
**1.08**	0.00	0.06	1.55	1.84
**0.76**	0.01	0.09	1.79	2.04
**0.51**	0.07	0.39	3.33	3.60

Mean absolute error (MAE) and root mean squared error (RMSE) for five collections of realistic synthetic data at varying levels of motif conservation. In these tests, the motif discovery phase of the algorithm was removed and the set of candidate models constructed as if the motif discovery algorithm had performed as well as possible at each candidate width.

We first note from Table 4 that the width predicted by MCOIN closely matches the true width in almost all cases; the error in the predicted width increases slightly as mean motif conservation is decreased. The E-values estimator initially matches MCOIN but quickly begins to underestimate motif width, leading to a much larger increase in the error in predicted width. MCOIN shows a clear performance advantage in terms of predicted width at all conservation levels.

Given that the widths predicted by MCOIN generally match the known width, it is unsurprising that the classification-based results (Table 3) match those in the case where the width is known. As noted above,*sSn* decreases with decreasing motif conservation. A similar, but less sharp, decrease is seen in*sPPV*. Although the E-values estimator slightly outperforms MCOIN in terms of*sSn* for the data collections with mean motif conservation of 1.08 bits/col and 0.76 bits/col (0.82 compared to 0.80 and 0.56 compared to 0.49, respectively), the corresponding values of*sPPV* are outperformed by MCOIN (0.93 compared to 0.79 and 0.89 compared to 0.71, respectively). Combining these results with the results presented in Table 3, this is likely a result of the E-values estimator choosing models at non-optimal widths which predict more sites overall at the expense of more false positive predictions.

### Realistic synthetic data

Subsequent evaluations use models computationally discovered by a MEME-based algorithm; the motif discovery phase of the algorithm is run as it would normally. Again, for each estimator, all candidate widths from*w*^∗^ - 4 to*w*^∗^ + 4 are tested. Results of evaluations on each of the five data collections are summarised in Tables 5 and 6.

**Table 5:** Realistic synthetic data: classification-based results

**Conservation**	**Known width (*w***^**∗**^)			**MCOIN (*w***^**∗**^** ± 4)**			**E-values (*w***^**∗**^** ± 4)**		
**(mean bits/col)**	***sSn***	***sPPV***	**AUC**	***sSn***	***sPPV***	**AUC**	***sSn***	***sPPV***	**AUC**
**2.00**	0.84	0.25	0.99	0.93	0.42	1.00	0.91	0.79	0.99
**1.49**	0.26	0.07	0.98	0.28	0.15	0.99	0.21	0.45	0.98
**1.08**	0.02	0.01	0.96	0.01	0.01	0.96	0.01	0.23	0.96
**0.76**	0.00	0.00	0.94	0.00	0.00	0.93	0.00	0.12	0.94
**0.51**	0.00	0.00	0.93	0.00	0.00	0.93	0.00	0.09	0.93

Mean site-level sensitivity (*sSn*), positive predictive value (*sPPV*) and area under the ROC curve (AUC) for five collections of realistic synthetic data at varying levels of motif conservation. In these tests, the motif discovery algorithm was allowed to run as it would normally.

**Table 6:** Realistic synthetic data: mean error in motif width

**Conservation**	**MCOIN (*w***^**∗**^** ± 4)**		**E-values (*w***^**∗**^** ± 4)**	
**(mean bits/col)**	**MAE**	**RMSE**	**MAE**	**RMSE**
**2.00**	1.60	2.06	1.80	2.28
**1.49**	1.59	2.08	2.46	2.82
**1.08**	1.97	2.42	2.16	2.51
**0.76**	2.38	2.74	1.84	2.22
**0.51**	2.38	2.71	1.95	2.32

Mean absolute error (MAE) and root mean squared error (RMSE) for five collections of realistic synthetic data at varying levels of motif conservation. In these tests, the motif discovery algorithm was allowed to run as it would normally.

We note that the results for predictions at the known width are generally lower than when the motif discovery phase of the algorithm was removed. These results illustrate the fact that the core motif discovery algorithm is far from perfect: even when the true motif width is known, classification-based results may be low. In all data collections, both MCOIN and the E-values estimator are shown to have a performance similar to or better than that at the known width in terms of classification-based measures. As noted by [12], this may be attributed to the fact that predicted sites are only required to overlap the known site by a quarter in order to be counted as a true positive.

As noted above, results for all three classification-based measures generally decrease as mean motif conservation also decreases (Table 5). At higher levels of motif conservation, MCOIN is shown to outperform the E-values estimator in terms of*sSn*. In this test, MCOIN generally chooses models which increase*sSn*, at the expense of*sPPV*. That is, MCOIN chooses models which tend to predict more false positive sites. While we would prefer to have few false results (that is, higher values for both*sSn* and*sPPV*) overall, it may be preferable to increase*sSn* at the expense of*sPPV*. For example, when searching for putative binding sites to be verified experimentally, it may be more useful to have more false positives than false negatives. The E-values estimator is shown to achieve a higher*sPPV* in all cases; this matches the findings of [12], where the E-values estimator was shown to achieve a slightly higher*sPPV* than other estimators on datasets containing human TFBS motifs. At higher levels of motif conservation, MCOIN is also shown to outperform the E-values estimator in terms of AUC.

While MCOIN generally matches the E-values estimator in terms of overall correctness based on AUC values, this does not represent the full picture. It follows from the above that an estimator may appear to perform well even if the chosen width does not match the true width [12]. Errors in the predicted width are presented in Table 6. We note from these results that the error in width predicted by both estimators generally increases as mean motif conservation is decreased. However, at higher levels of motif conservation, MCOIN outperforms the E-values estimator using both error measures.

### ***E. coli* and prokaryotic ChIP data**

MCOIN was then evaluated in the same manner using diverse prokaryotic TFBS sequences as described above. The results of this evaluation are summarised in Tables 7, 8, 9, 10.

**Table 7:** ***E. coli ***data: classification-based results

Conservation	Known width (***w***^***∗***^)			MCOIN (***w***^**∗**^** ± 4)**			E-values (***w***^**∗**x^** ± 4)**		
(mean bits/col)	***sSn***	***sPPV***	**AUC**	***sSn***	***sPPV***	**AUC**	***sSn***	***sPPV***	**AUC**
**‘high’ (1.36)**	0.81	0.22	0.96	0.72	0.29	0.96	0.70	0.17	0.95
**‘low’ (0.78)**	0.63	0.41	0.96	0.69	0.51	0.98	0.66	0.32	0.97
**overall (1.13)**	0.74	0.30	0.96	0.71	0.38	0.96	0.68	0.23	0.96

Mean site-level sensitivity (*sSn*), positive predictive value (*sPPV*) and area under the ROC curve (AUC) for 20 datasets created using real*E. coli* data.

**Table 8:** ***E. coli ***data: mean error in motif width

Conservation	MCOIN (***w***^**∗**^** ± 4)**		E-values (***w***^**∗**^*** ± 4)***	
(mean bits/col)	MAE	RMSE	MAE	RMSE
‘high’ (1.36)	2.08	2.43	2.92	3.12
‘low’ (0.78)	1.75	2.06	3.00	3.20
overall (1.13)	1.95	2.29	2.95	3.15

Mean absolute error (MAE) and root mean squared error (RMSE) for 20 datasets created using real E. coli data.

**Table 9:** Prokaryotic ChIP data: classification-based results

Conservation	Known width (***w***^**∗**^)			MCOIN (***w***^**∗**^*** ± 4)***			E-values (***w***^**∗**^** ± 4)**		
(mean bits/col)	***sSn***	*sPPV*	**AUC**	***sSn***	***sPPV***	**AUC**	***sSn***	***sPPV***	**AUC**
**0.99**	0.75	0.67	0.99	0.75	0.68	0.99	0.73	0.67	0.99

Mean site-level sensitivity (*sSn*), positive predictive value (*sPPV*) and area under the ROC curve (AUC) for 9 datasets created using real prokaryotic data determined through ChIP experiments. The datasets used are summarised in Table 2.

**Table 10:** Prokaryotic ChIP data: mean error in motif width

**Conservation**	**MCOIN (*w***^**∗**^** ± 4)**		**E-values (*w***^**∗**^*** ± 4)***	
**(mean bits/col)**	**MAE**	**RMSE**	**MAE**	**RMSE**
**0.99**	1.44	1.86	2.33	2.73

Mean absolute error (MAE) and root mean squared error (RMSE) for 9 datasets created using real prokaryotic data determined through ChIP experiments. The datasets used are summarised in Table 2.

The heterogeneity of the motifs in both data collections may suggest that results for these datasets could be equally varied. However, both MCOIN and the E-values estimator are reasonably robust in terms of predicted sites (Tables 7 and 9). While the*sSn* results for the low conservation group in the*E. coli* data collection are lower than that for the high conservation group,*sPPV* increases with decreasing motif conservation. This is a result of the smaller set of predicted sites containing fewer false positive results and can be attributed to the small number of datasets tested. When combined, the reduction in the number of false positive predictions and the consistently high AUC values suggest that models are chosen where true motif occurrences are predicted with greater confidence. For both the*E. coli* and prokaryotic ChIP data collections, MCOIN outperforms the E-values estimator in terms of classification-based results. The prokaryotic ChIP data collection shows a slight improvement in*sSn* and*sPPV* values (Table 9); this improvement is greater in the*E. coli* data collection (Table 7). It is also noted that the classification-based results for MCOIN better match those at the known width than the results of the E-values estimator.

Tables 8 and 10 present the mean error in motif width based on both data collections. MCOIN is shown to outperform the E-values estimator for both data collections. Although the mean error in motif width for models predicted by MCOIN appears to decrease with decreasing motif conservation in the*E. coli* data collection, this is explained by the small number of datasets tested. The small number of datasets tested also accounts for the fact that the error in motif widths predicted by the E-values estimator is relatively high for both real data collections, given the results previously obtained on realistic synthetic data.

**Figure 3:**
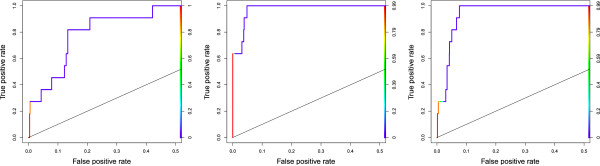
***E. coli*: RcsB motif ROC curves.** ROC curves (plotted for 0≤sFPR≤0.5) for the most likely E.coli RcsB motif, as chosen using the known width (left), MCOIN (centre) and E-values based estimator (right). The curve colour illustrates the threshold of p(Z_*i,j*_ = 1|X_*i,j*_,θ), from 1.0 (red) to 0.0 (blue). Although MCOIN and the E-values estimator both underestimate the known motif width, site-level predictions are improved as the true motif is relatively weakly discovered at the true width. Performance in terms of AUC may be increased by choosing stronger and/or unshifted motif models at non-optimal widths. MCOIN displays improvement over the known motif width and the E-values estimator in all three classification performance measures.

We noted earlier that performance in terms of AUC may be improved by choosing a better motif model at a non-optimal width. The*E. coli* RcsB motif provides an example of this (Figure 3 illustrates some of the observations made here). At the true width (*w*^∗^ = 14bp), the motif is discovered relatively poorly (*sSn* = 0.27,*sPPV* = 0.21, AUC = 0.88). Both MCOIN and the E-values based estimator improve AUC by choosing models at shorter widths. The E-values estimator chooses the model at*w*^∗^ - 2 (*sSn* = 0.27,*sPPV* = 0.09, AUC = 0.97) and MCOIN chooses the model at*w*^∗^ - 4 (*sSn* = 0.64,*sPPV* = 0.39, AUC = 0.99). MCOIN displays improvement in all measures; it may be concluded that, although the chosen width is not the true motif width, the model chosen by MCOIN is a better model overall. Similar results are noted in the CaiF, FruR and PurR motifs in the*E. coli* data collection and the*B. subtilis* Spo0A motif in the prokaryotic ChIP data collection. As noted above, the model at the optimal width need not be the closest match to the biologically known motif. The results presented in Figure 3 also show that the model chosen by MCOIN gives more predictions at higher values of*pZ*_*ij*_ = 1|*X*_*i*,*j*_θ), compared to the model chosen by the E-values estimator and the model at the true width. Similar results are noted for some other*E. coli* motifs, although this cannot be guaranteed for all motifs.

**Figure 4:**
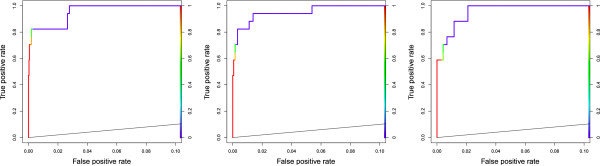
*E. coli*: GntR motif ROC curves. ROC curves (plotted for 0≤sFPR≤0.1) for the most likely E.coli GntR motif, as chosen using the known width (left), MCOIN (centre) and E-values based estimator (right). The curve colour illustrates the threshold of *p*(*Z*_*i,j*_ = 1|X_*i,j*_,θ), from 1.0 (red) to 0.0 (blue). All three estimators predict the GntR motif much better than expected, considering the low conservation of the motif and the results of the experiments using realistic synthetic data.

Comparing Tables 7 and 9 to Table 5, MCOIN is shown to give excellent classification-based results (particularly on the prokaryotic ChIP datasets) given the overall mean motif conservation and the results on realistic synthetic data. This is due to the conservation of individual positions within each motif: while the conservation of positions in each synthetic motif is uniform and independent, this pattern of conservation is not mirrored in real TFBS motifs. Analysis of the previously characterised motifs used in this study indicates that motifs with low mean conservation may have several positions which are very well or even perfectly conserved. This matches well with previous studies (e.g. [30]), which propose that the conservation of a given motif position is correlated with the conservation of surrounding motif positions, producing clusters of well-conserved positions, which may aid TFBS motif discovery algorithms. This phenomenon is clear in a number of E. coli motifs, particularly GntR (Figure 4), which has a mean conservation of 0.74 bits/col; the synthetic data results suggest relatively low values of sSn and sPPV for this motif. However, the GntR motif has a cluster of reasonably well-conserved positions, with a maximum conservation of 1.61 bits/col and is discovered well at the known width (sSn = 0.82, sPPV = 0.70, AUC = 0.99), with similar results for both the MCOIN and E-values based estimators (sSn = 0.71, sPPV = 0.71, AUC = 0.99 and sSn = 0.71, sPPV = 0.48, AUC = 1.00, respectively).

## Conclusions

Determining the width of a TFBS motif is an important and challenging problem with direct relevance to computational motif discovery. MCOIN is a novel heuristic for determining the width of a motif, based on motif containment and information content. Results of tests on two data collections of previously characterised prokaryotic motifs show that MCOIN outperforms the E-value of the resulting multiple alignment (currently the most widely used estimator) as a predictor of motif width, using mean absolute error and root mean squared error. MCOIN is also shown to choose models which improve the overall correctness of predicted motif sites, based on site-level sensitivity, positive predictive value and the area under the ROC curve.

MCOIN also has a clear advantage over methods based on cross-validation with limited numbers of folds, as all available data is used for motif discovery, improving discovery results. Further, the results of experiments which removed the motif discovery phase of the algorithm show that, as the performance of this phase improves, the performance of MCOIN as a predictor of motif width also improves: as the discovered model becomes stronger and better models the true motif, the error in the width estimated by MCOIN will decrease.

## Competing interests

The authors declare that they have no competing interests.

## Authors’ contributions

SA, BW and AMK designed the study. AMK performed the research and drafted the manuscript. All authors read and approved the final manuscript.

## Supplementary Material

Additional file 1 Summary of experimental evidence for each TFBS sequence used in the ***E. coli ***data collection. Click here for file
